# Supplementary material for: Complement-mediated killing of Escherichia coli by mechanical destabilization of the cell envelope
Source: EMBO J. 2024 Oct 14;43(23):6152–60. doi: 10.1038/s44318-024-00266-3 (PMC11612287; doi:10.1038/s44318-024-00266-3)
Supplement: Supplementary file 1 — Appendix [file 44318_2024_266_MOESM1_ESM.pdf]

Complement-mediated killing of *Escherichia coli* by mechanical destabilization of the cell envelope

Georgina Benn, Christian Bortolini, David M. Roberts, Alice L. B. Pyne,

Séamus Holden, and Bart W. Hoogenboom

Appendix

Table of Contents

p. 2 - Appendix Figure S1. MAC formation is highly variable between cells exposed to the same concentrations of complement proteins.

p. 3 - Appendix Figure S2. MAC are distributed evenly over the surface of a single cell.

p. 4 - Appendix Figure S3. MACs do not show significant clustering.

p. 5 - Appendix Figure S4. Inner membrane permeabilization correlates with major mechanical disruption.

p. 6 - Appendix Figure S5. Further examples of major disruption of the cell envelope precedes cell lysis.

p. 7 - Appendix Figure S6. Major outer membrane disruption or destabilization is not a generic feature of cell lysis.

p. 7 - Appendix Figure S7. Defect formation initiated at MAC pore.

p. 8 - Appendix Figure S8. Complement exposure leads to extensive and progressive defect formation at the bacterial surface.

p. 9 - Appendix Figure S9. Defects do not propagate after bacterial cell death.

p. 10 - Appendix Figure S10. Changes in height and surface stiffness for individual BL21(DE3) *E. coli* cells exposed to complement.

p. 11 - Appendix Figure S11. Changes in height and surface stiffness for individual MG1655 *E. coli* cells exposed to complement.

p. 12 - Appendix Figure S12. C8 and C9 are required for complement to induce changes in height and surface stiffness of *E. coli*.

p. 13 - Appendix Figure S13. Prior bacterial swelling and surface stiffening are not generic features of cell lysis.

p. 14 - Appendix Figure S14. Representative images of SYTOX^{TM} and membrane-stained *E. coli* MG1655.

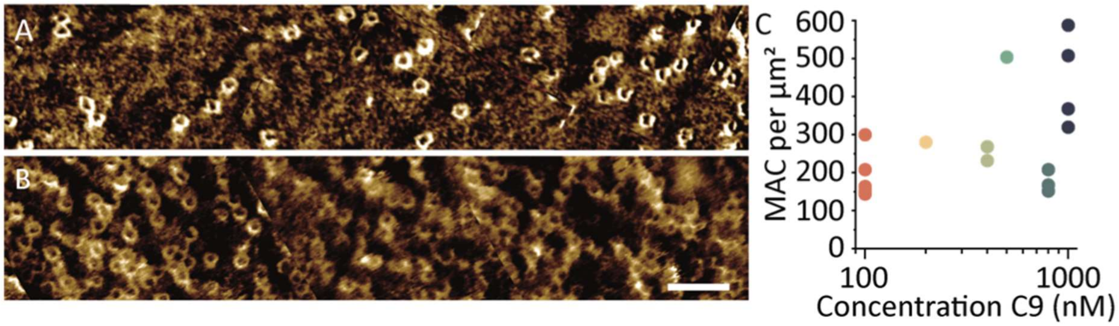

Appendix Figure S1. MAC formation is highly variable between cells exposed to the same concentrations of complement proteins.

(A-B) Whole-cell phase images show that the overall densities of MACs on the BL21(DE3) *E. coli* surface were highly varied. Some cells had (A) sparse MACs, whereas some had (B) dense packing of MACs. (C) The number of MACs in the surface was highly varied between samples, with no consistent increase for C9 concentrations ranging from 100 to 1000 nM, suggesting that the C9 concentration was not the limiting step in these experiments. Colour scale: (A) 2 deg, (B) 3.25 deg. Scale bar: 100 nm. Data refer to 16 different live cells in independent experiments.

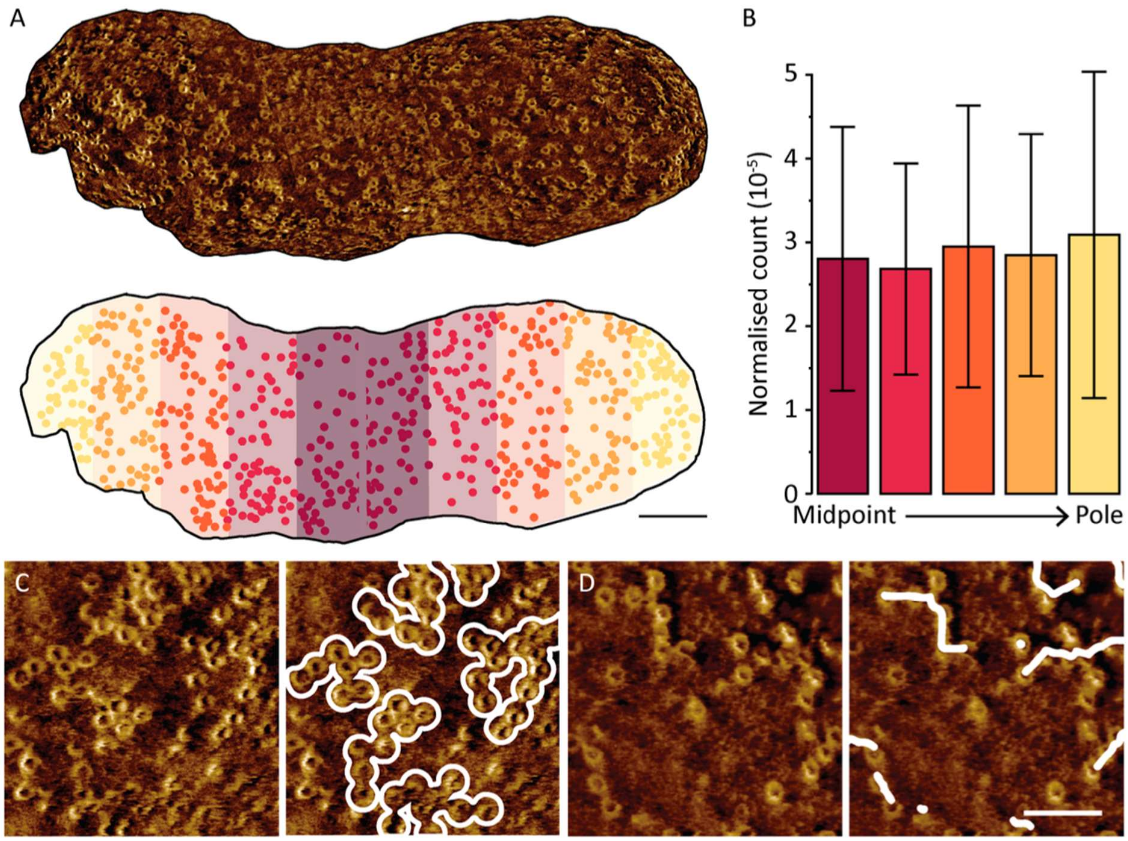

Appendix Figure S2. MAC are distributed evenly over the surface of a single cell.

(A) Top, AFM phase images were used to identify MACs and the cell midpoint. Bottom, the surface was divided into 10 regions from midpoint to pole. The numbers of MACs in each region were counted and the counts normalised to the area of each region. (B) This analysis showed the density of MACs (per nm^2) did not change across the cell surface for n = 5 different cells. Error bars indicate standard deviations (C) At first sight, MACs appeared to cluster in groups when density was high, leaving regions of bare membrane. The right image duplicates the left image, but with MAC clusters marked in white. (D) When the density was lower, MACs appeared to form in lines. The right image duplicates the left image but with branches of MACs marked in white. Colour scale: (A) 3.25 – 5 deg (C-D) 3.5 deg. Scale bars: (A) 200 nm and (C-D) 100 nm.

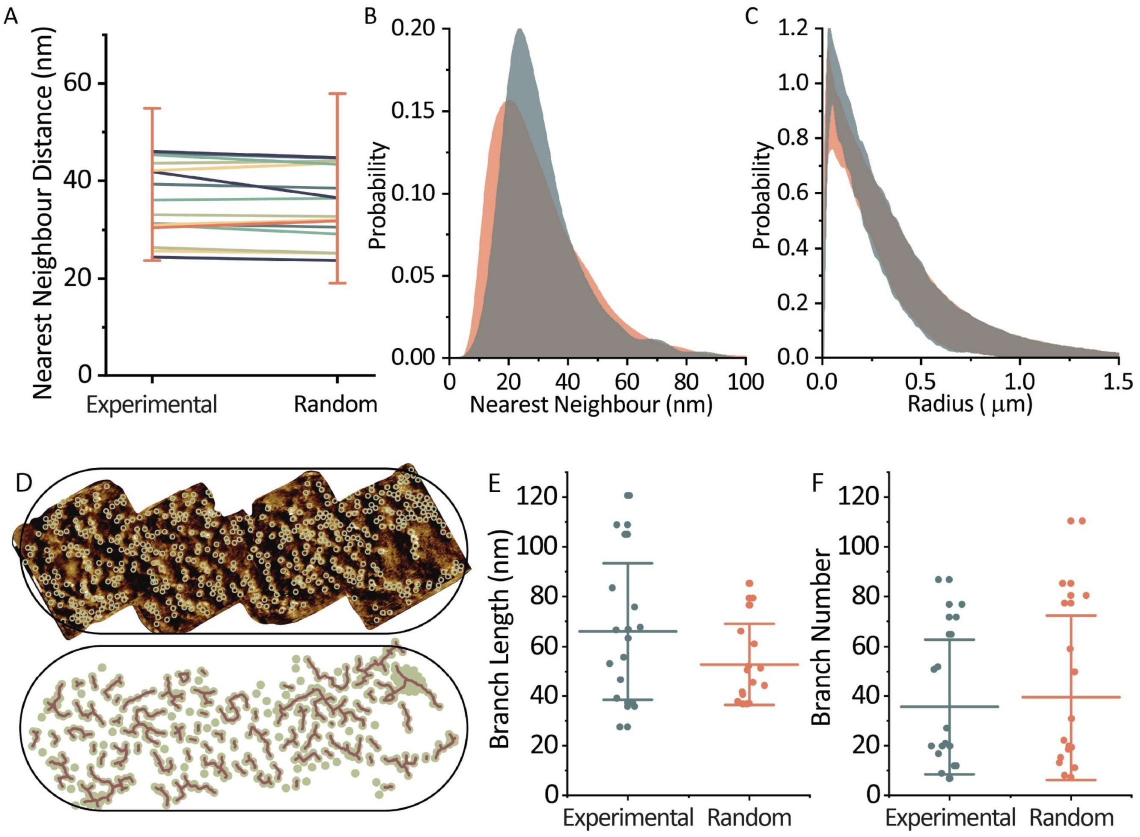

Appendix Figure S3. MACs do not show significant clustering.

From the comparison of experimental data on MAC locations with locations that were randomly generated over the same areas, the randomness of MAC distributions can be quantified. (A) Mean nearest-neighbour (MAC-MAC) distances for different samples. Error bars show typical standard deviations, only one sample standard deviation is shown for clarity. (B) Histogram of all nearest neighbours show similar distributions for real (green) and random (pink) data. (C) Radial distributions show no difference between long-distance clustering of MACs from random points. Bands corresponding to real (green) and random (pink) standard deviations are shown. (D) Chains of MACs were investigated by picking MAC points, dilating them to circles that overlap when nearby, skeletonising the resulting shape and finding the longest branch. This showed that (E) experimental and randomly generated MAC positions led to branch lengths of approximately equal size. (F) The number of branches in experimental data was also not significantly different from the random case. Data are from MACs on 16 cells.

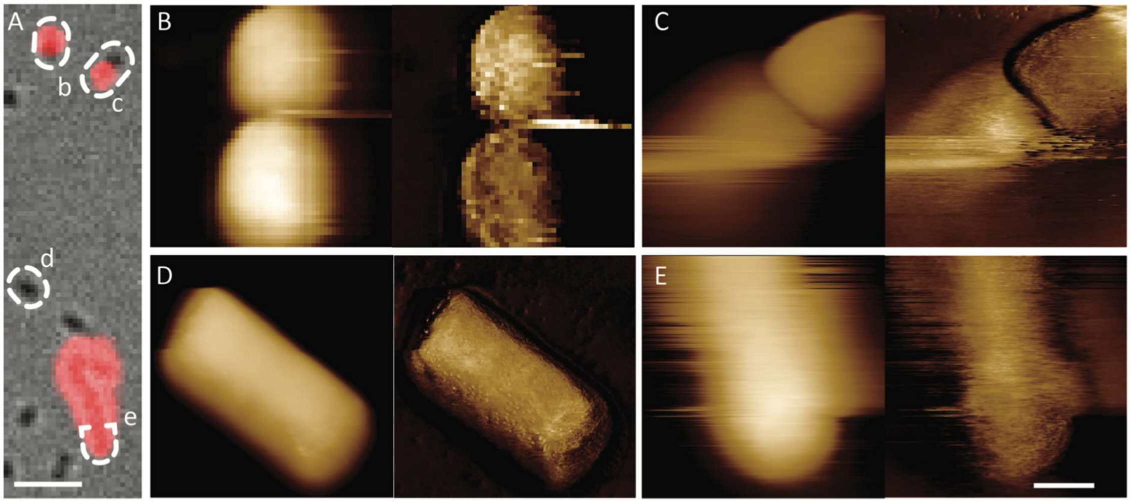

Appendix Figure S4. Inner membrane permeabilization correlates with major mechanical disruption.

(A) Merge of brightfield (grey) and SYTOX™ (red) images with bacteria imaged in B-E indicated with corresponding letters. (B-E) Height (left) and phase (right) images show that, when cells are dead (both cells in B, the bottom cell in C and the cell in E), their outer membranes become less stable, demonstrated by streaks in images as the AFM tip pulls the membrane around. However, the membranes of live cells (top cell in C and cell in D) are stable and intact. Vertical height scale is 600 nm and phase scales are (B) 10 deg, (C) 20 deg, (D) 15 deg and (E) 15 deg. Scale bars are (A) 5 µm and (B-E) 500 nm.

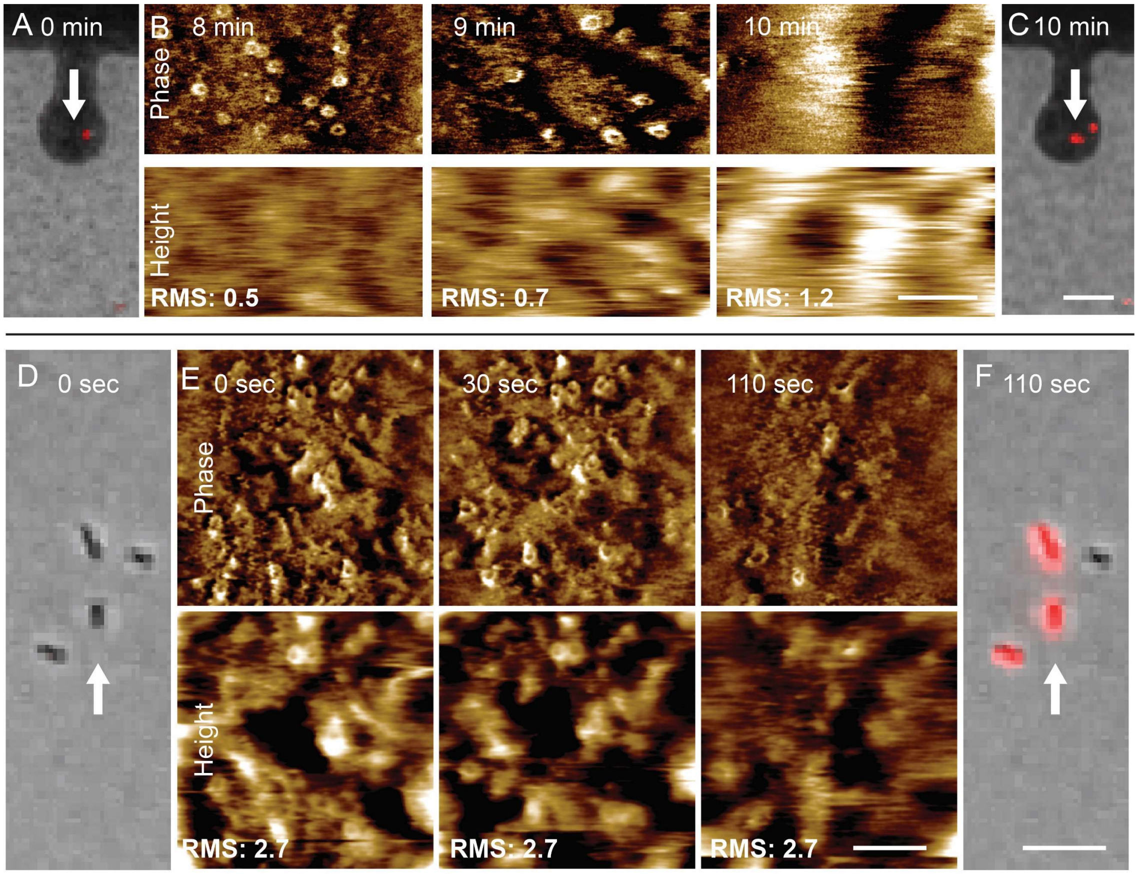

Appendix Figure S5. Further examples of major disruption of the cell envelope precedes cell lysis. (A) Merge of brightfield (grey) and SYTOX™ (red) images of BL21(DE3) *E. coli* at the beginning of imaging in B (= 0 min). (B) AFM phase and height images of the cell that is marked by the white arrow in A, as a function of time, showing loss of contrast and roughening (quantified by an increase in RMS from 0.7 to 1.2) at approximately 9 minutes. (C) The cell is SYTOX™ positive (red) when next inspected for inner membrane permeation. (D) Merge of brightfield (grey) and SYTOX™ (red) images of BL21(DE3) *E. coli* at the beginning of imaging in E (= 0 sec). (E) AFM phase and height images of the cell that is marked by the white arrow in D, as a function of time, showing loss of contrast and rearrangement of MACs at approximately 100 seconds. (F) The cell is SYTOX™ positive (red) when next inspected for inner membrane permeation. Colour scale (see inset Figure 1C): 1.5 deg and 5 nm (B), 3 deg and 10 nm (E). Scale bars: 5 µm (A, C, D and F) and 100 nm (B and E). Time points are relative to the initial image (A,D).

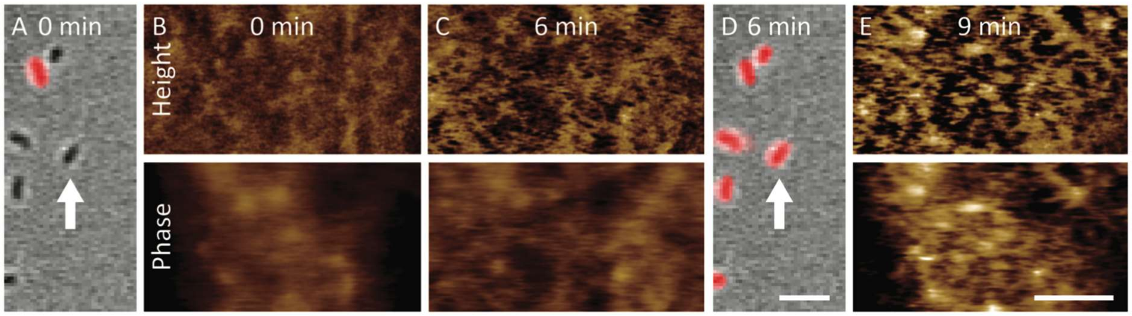

Appendix Figure S6. Major outer membrane disruption or destabilization is not a generic feature of cell lysis.

(A and D) Merges of brightfield (grey) and SYTOX™ (red) images show the imaged bacterium, indicated by the white arrow. (B, C and E) Phase and height images show that the outer membrane remains intact throughout melittin killing as the high resolution and roughening surface features are still visible. The roughness is reflected by an increase in RMS from 5 nm in B to 13 nm in C and 40 nm in E. Colour scales are 2 deg and 10 nm. Scale bars are (A and D) 5 µm and (B, C and E) 100 nm. Time points are relative to the initial image.

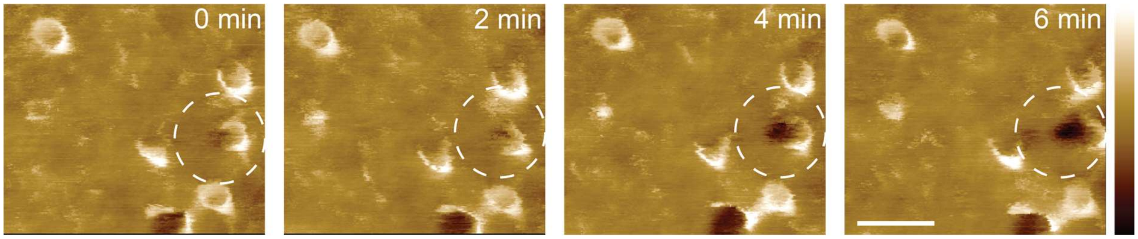

Appendix Figure S7. Defect formation initiated at MAC pore.

Sequence of AFM (height) images of BL21(DE3) *E. coli*, taken at 2 minutes per frame, showing a defect (black hole, marked by a dashed circle) initiating at a single MAC assembly. Colour scale (bar shown on right hand side) is 20 nm. Scale bar is 50 nm**.**

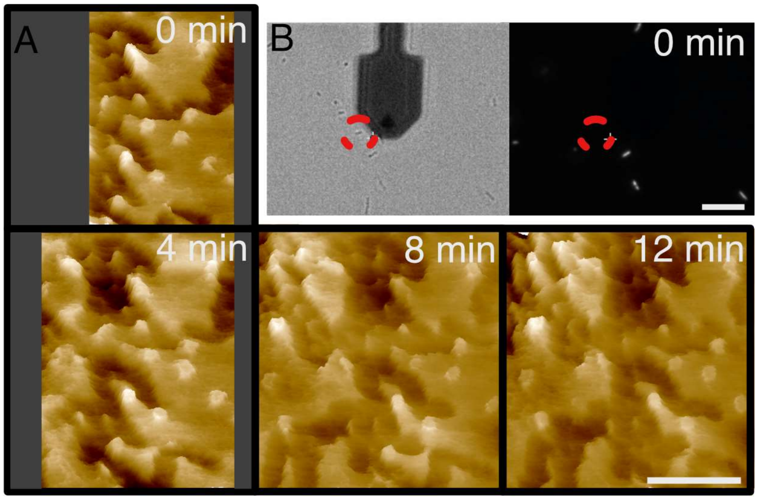

Appendix Figure S8. Complement exposure leads to extensive and progressive defect formation at the bacterial surface.

(A) Sequence of AFM (height) images of BL21(DE3) *E. coli*, cropped and aligned from Movie EV2, showing MAC pores and larger (> 50 nm wide) defects in the outer membrane. Times are referenced with respect to the first recorded high-resolution image (0 min). (B) Brightfield (left) and SYTOX™ fluorescence (right) microscopy images of the bacteria in the AFM experiments, with red dashed ellipses indicating the (SYTOX™ negative) cell on which the AFM sequence was recorded. The dark paddle is the AFM cantilever. Colour scale: 30 nm; scale bars: 100 nm (A) and 10 µm (B).

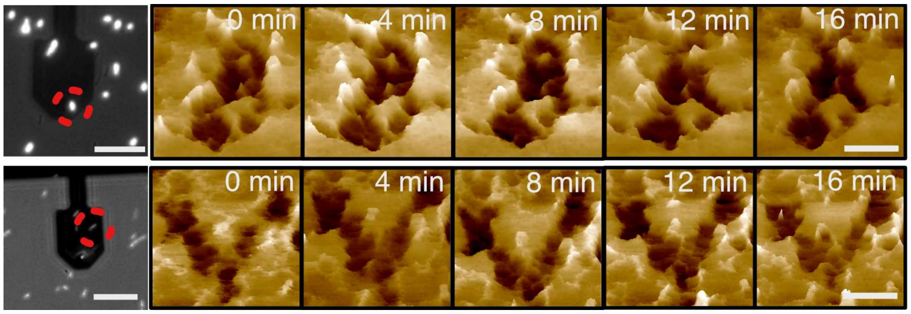

Appendix Figure S9. Defects do not propagate after bacterial cell death.

Two examples (top and bottom row) of high-resolution SYTOX™ fluorescence (first frame) and sequence of AFM images (at surface of cell marked with red dashed circle in first frame) on cells with compromised inner membranes (positive SYTOX™ signal). Colour scale: 30 nm; scale bars: 10 µm (fluorescence images); 100 nm (AFM).

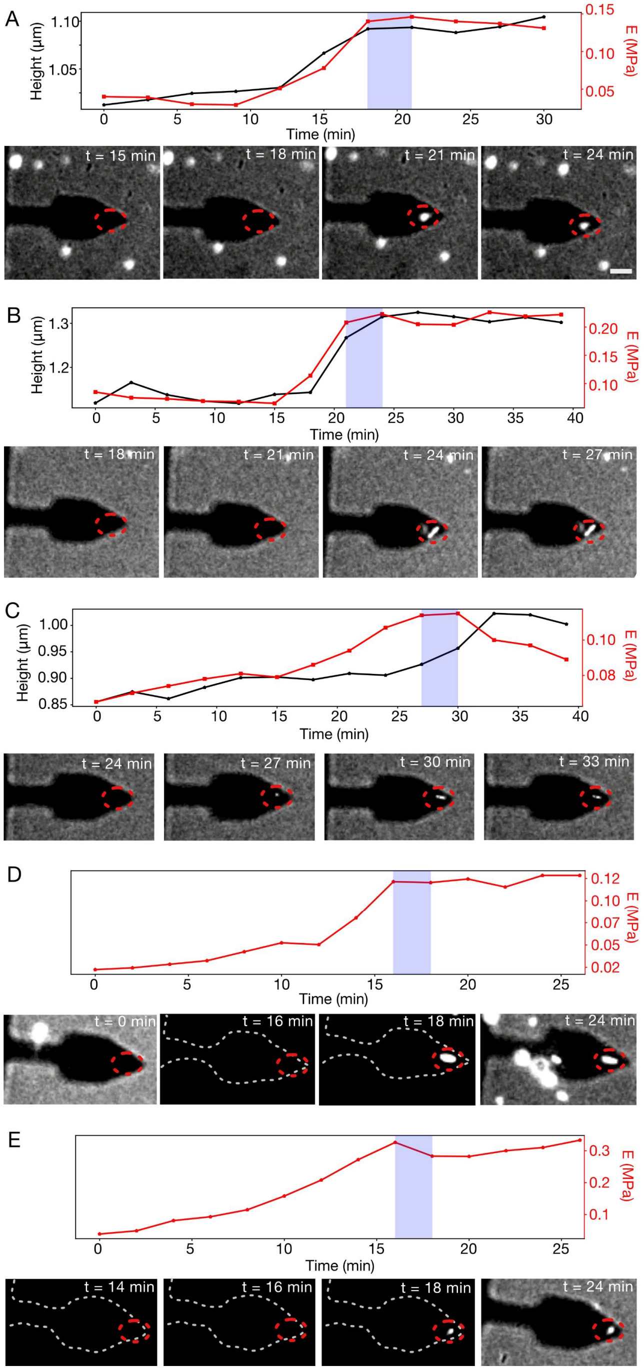

Appendix Figure S10. Changes in height and surface stiffness for individual BL21(DE3) *E. coli* cells exposed to complement.

(A-E) Changes for *n* = 5 individual cells of BL21(DE3) *E. coli*, as summarized in Figure 4, with according SYTOX™ fluorescence data. Vertical, blue-shaded bands indicate the time at which the cell first stained SYTOX^{TM} positive, indicative of inner membrane permeation, as shown by the fluorescence microscopy images. The measured cells are marked by red, dashed circles. Scale bar: 10 µm.

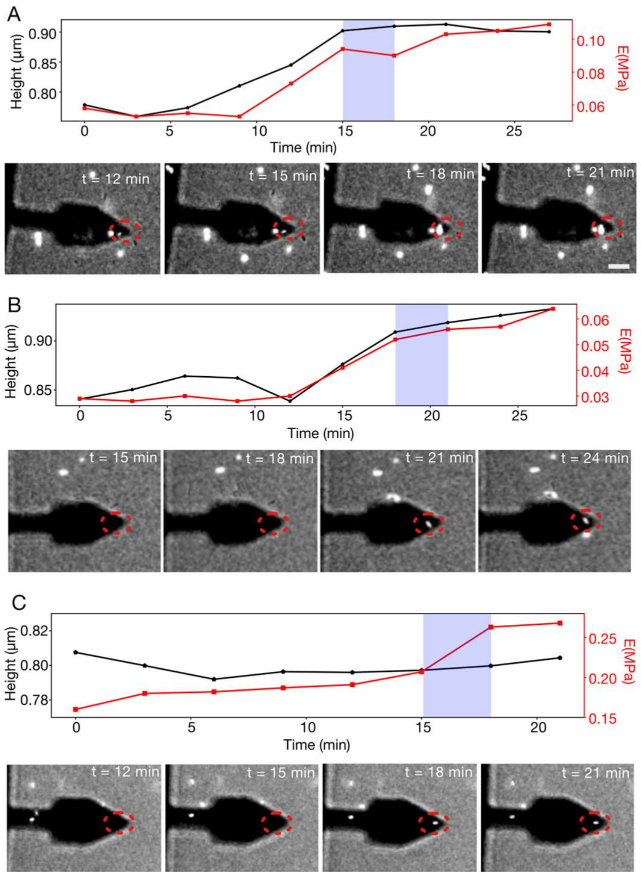

Appendix Figure S11. Changes in height and surface stiffness for individual MG1655 *E. coli* cells exposed to complement.

(A-C) Changes for *n* = 3 individual cells of MG1655 *E. coli*, as summarized in Figure 4, with according SYTOX™ fluorescence data. Vertical, blue-shaded bands indicate the time at which the cell first stained SYTOX™ positive, indicative of inner membrane permeation, as shown by the fluorescence microscopy images. The measured cells are marked by red, dashed circles. Scale bar: 10 µm.

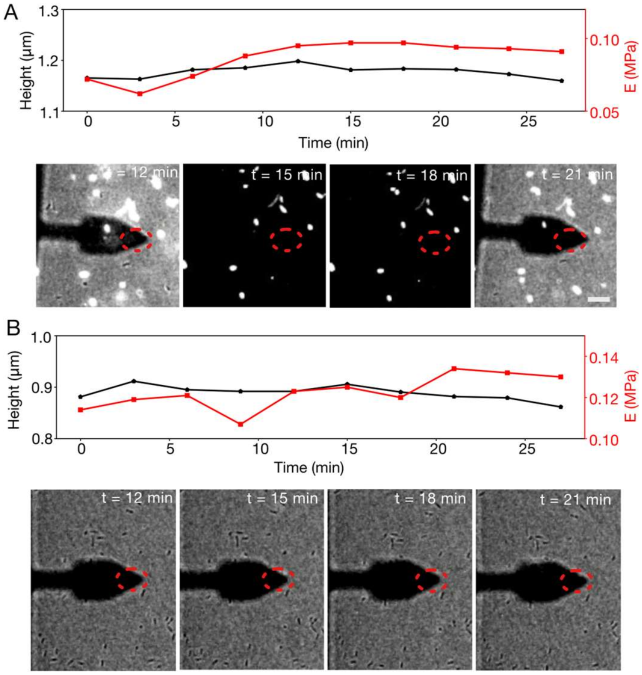

Appendix Figure S12. C8 and C9 are required for complement to induce changes in height and surface stiffness of *E. coli*.

Negative control experiments for the results shown in Figure 4, Appendix Figures S10, S11). BL21(DE3) (A) and MG1655 (B) subjected to the same protocol as in (Figure 4, Appendix Figures S10, S11), but without addition of C8 and C9. Vertical, blue-shaded bands indicate the time at which the cell first stained SYTOX™ positive, indicative of inner membrane permeation, as shown by the fluorescence microscopy images .Over timescales investigated, no cell death or noticeable increase in cell size or surface stiffness were observed.

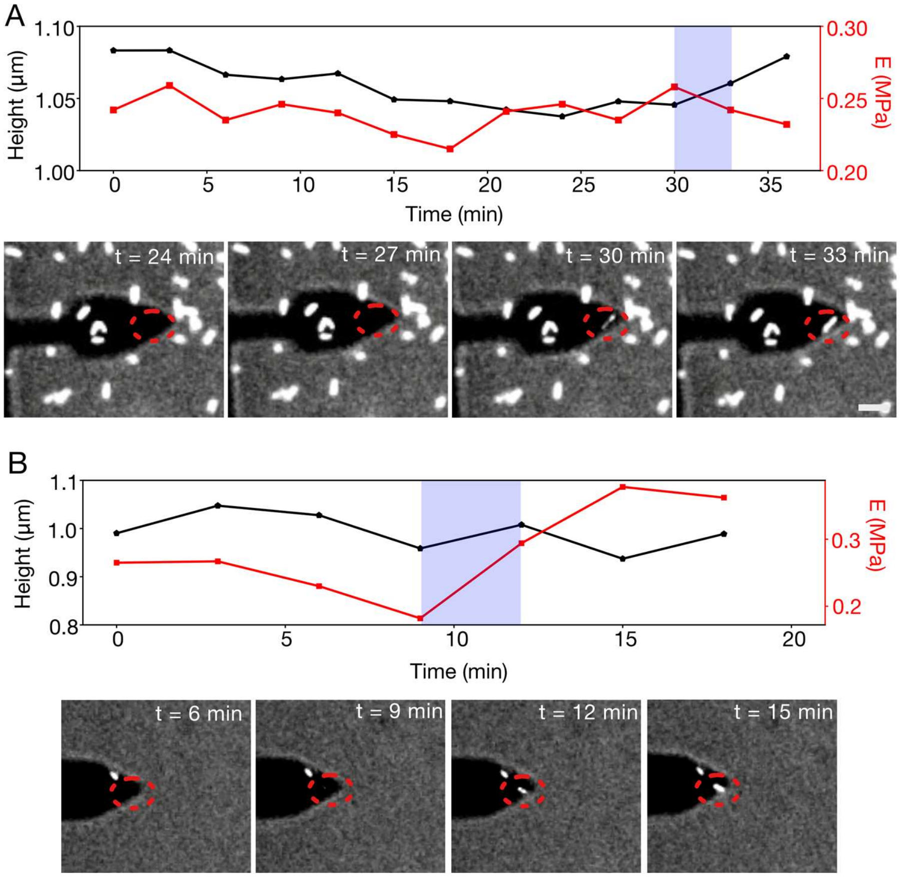

Appendix Figure S13. Prior bacterial swelling and surface stiffening are not generic features of cell lysis.

(A, B) Two negative control experiments for the results shown in Figure 4, Appendix Figures S10, S11. BL21(DE3) *E. coli* is exposed to and killed by Melittin, as shown by the SYTOX^{TM} staining, but show no clear trend in size or stiffness increase.

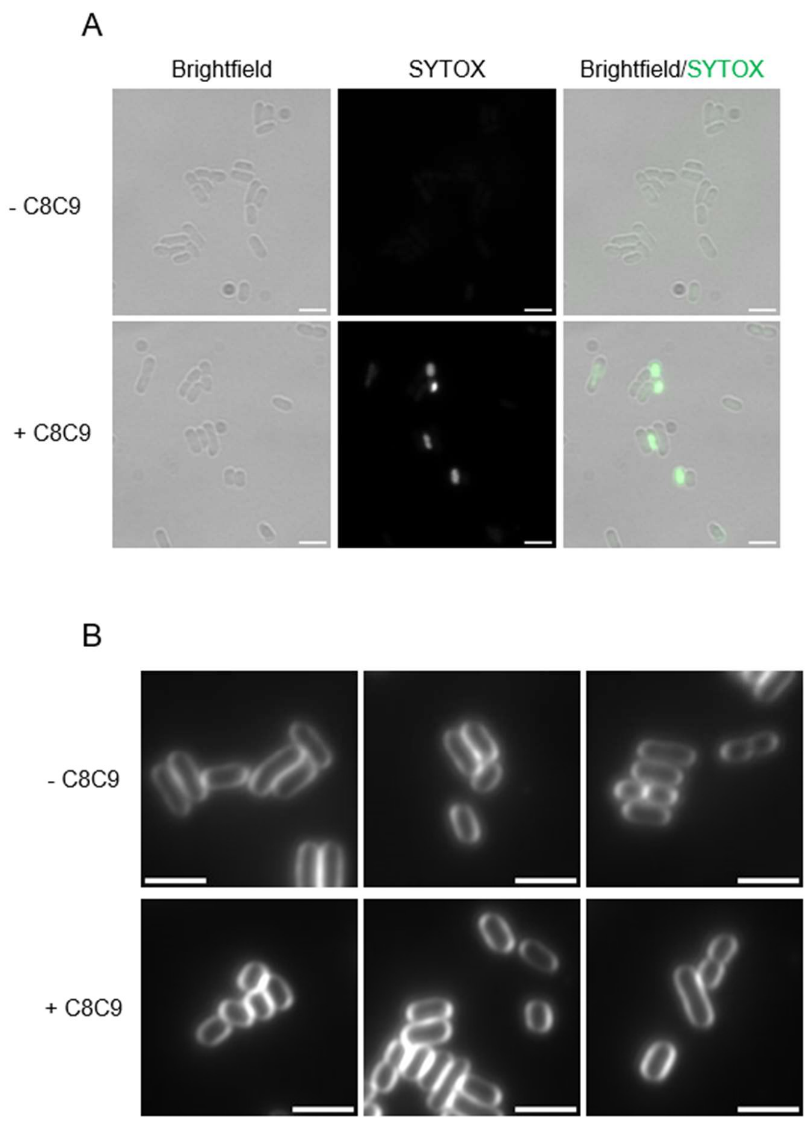

Appendix Figure S14. Representative images of SYTOX™ and membrane-stained *E. coli* MG1655. (A) Representative images of SYTOX™ stained *E. coli* MG1655 cells ± C8C9 treatment for 15 min, as used for quantitative analysis presented in Figure 4E. (B) Representative images of FM5-95 stained *E. coli* MG1655 cells treated ±C8C9, as used for quantitative analysis presented in Figure 4F. Scale bars: 3 µm.
